# Supplementary material for: Antithrombotic drugs for cardiovascular risk reduction in patients with lower limb peripheral arterial disease: protocol for a systematic review and network meta-analysis of randomised controlled trials
Source: BMJ Open. 2023 Aug 10;13(8):e072355. doi: 10.1136/bmjopen-2023-072355 (PMC10423790; doi:10.1136/bmjopen-2023-072355)
Supplement: Supplementary data [file bmjopen-2023-072355supp001.pdf]

Supplemental Material

Antithrombotic medications currently named in the British National Formulary are aspirin, clopidogrel, dipyridamole, prasugrel, ticagrelor, cangrelor, warfarin sodium, acenocoumarol, phenindione, apixaban, edoxaban, rivaroxaban, dabigatran etexilate, heparin (unfractionated), dalteparin sodium, enoxaparin sodium, tinzaparin sodium, danaparoid sodium, argatroban monohydrate, bivalirudin, epoprostenol, and fondaparinux.ubmission
