# Supplementary material for: Antithrombotic drugs for cardiovascular risk reduction in patients with lower limb peripheral arterial disease: protocol for a systematic review and network meta-analysis of randomised controlled trials
Source: BMJ Open. 2023 Aug 10;13(8):e072355. doi: 10.1136/bmjopen-2023-072355 (PMC10423790; doi:10.1136/bmjopen-2023-072355)
Supplement: Author's manuscript [file bmjopen-2023-072355.draft_revisions.pdf]

BMJ Open is committed to open peer review. As part of this commitment we make the peer review history of every article we publish publicly available.

When an article is published we post the peer reviewers' comments and the authors' responses online. We also post the versions of the paper that were used during peer review. These are the versions that the peer review comments apply to.

The versions of the paper that follow are the versions that were submitted during the peer review process. They are not the versions of record or the final published versions. They should not be cited or distributed as the published version of this manuscript.

BMJ Open is an open access journal and the full, final, typeset and author-corrected version of record of the manuscript is available on our site with no access controls, subscription charges or pay-per-view fees (http://bmjopen.bmj.com).

If you have any questions on BMJ Open's open peer review process please email info.bmjopen@bmj.com

BMJ Open

Antithrombotic drugs for cardiovascular risk reduction in patients with lower limb peripheral arterial disease: protocol for a systematic review & network meta-analysis of randomised controlled trials

Journal:	*BMJ Open*
Manuscript ID	bmjopen-2023-072355
Article Type:	Protocol
Date Submitted by the Author:	01-Feb-2023
Complete List of Authors:	Sidebottom, David; St George's University of London, Molecular and Clinical Sciences Research Institute; St George's University Hospitals NHS Foundation Trust, St Georges Vascular Institute<br>Huang, Chao; Hull York Medical School<br>Carradice, Daniel; Hull York Medical School; Hull University Teaching Hospitals NHS Trust<br>Holt, Peter; St George's University of London, Molecular and Clinical Sciences Research Institute; St George's University Hospitals NHS Foundation Trust, St George's Vascular Institute<br>John-Pierre, Karen; St George's University of London, NHS Liaison Librarian<br>Roy, Iain; St George's University of London, Molecular and Clinical Sciences Research Institute; St George's University Hospitals NHS Foundation Trust, St George's Vascular Institute
Keywords:	Anticoagulation < HAEMATOLOGY, Vascular medicine < INTERNAL MEDICINE, Vascular surgery < SURGERY

SCHOLARONE™ Manuscripts

Protocol for a network meta-analysis

Antithrombotic drugs for cardiovascular risk reduction in patients with lower limb peripheral arterial disease: protocol for a systematic review & network meta-analysis of randomised controlled trials

Short title: Protocol for a NMA of antithrombotics for PAD

David B. Sidebottom *Academic Foundation Doctor* [1 2]

Chao Huang *Senior Lecturer in Statistics* [3]

Daniel Carradice *Senior Lecturer in Vascular and Endovascular Surgery* [3 4]

Peter Holt *Professor of Vascular Surgery* [1 2]

Karen John-Pierre *NHS Liaison Librarian* [1]

Iain N Roy *Hon. Senior lecturer in Vascular Surgery* [1 2*]

Affiliations

[1] St George's, University of London, London, United Kingdom

[2] St George's University Hospitals NHS Foundation Trust, London, United Kingdom

[3] Hull York Medical School, United Kingdom

[4] Hull University Teaching Hospitals NHS Trust, United Kingdom

[*]Address for correspondence

Mr Iain N Roy, Molecular and Clinical Sciences Research Institute, St. George's, University of London

E-mail: iroy@sgul.ac.uk

Word count: 3586 / 4000

Sponsor: St George's, University of London.

Role of the funder/sponsor: The funder peer reviewed but did not have any other role in the design, preparation, or writing of this protocol. The authors had full autonomy in all aspects of protocol development.

For peer review only - http://bmjopen.bmj.com/site/about/guidelines.xhtml

Copyright statement: This research was funded in whole, or in part, by NIHR (NIHR204123). For the purpose of open access, the author has applied a Creative Commons Attribution (CC BY) licence to any Author Accepted Manuscript version arising from this submission.'

For peer review only

For peer review only - http://bmjopen.bmj.com/site/about/guidelines.xhtml

Abstract [297 / 300 words]

Introduction

The optimal antithrombotic regimen to reduce the risk of vascular events in patients with peripheral arterial disease (PAD) is contentious. This systematic review and network meta-analysis aims to define the relative efficacy and risks of previously investigated antithrombotic medication regimens in preventing major cardiovascular events, vascular limb events, and mortality in patients with PAD.

Methods and analysis

A peer-reviewed, systematic search will be executed in English on Medline, Embase, Cochrane (CENTRAL), Web of Science, and Google Scholar databases in late 2022. The World Health Organisation International Clinical Trials Registry platform will also be searched for ongoing trials. Abstracts will be screened independently by two researchers for randomised controlled trials meeting the review criteria. All associated publications including the study protocol will be sought and evaluated together against pre-specified inclusion/exclusion criteria. Two researchers will extract the data into a pre-piloted extraction form. Risk of bias assessments will be performed using the Cochrane “Risk of Bias 2” criteria by individuals with domain expertise. All differences will be resolved by consensus or a third individual for ties.

Included trials will be summarised. A network meta-analysis will be performed, subject to checks of assumptions. Both primary and secondary outcomes will be analysed on a whole network basis. Pairwise comparisons and league tables will be produced. Pre-specified subgroup analyses will include sex, ethnicity, disease status, conservative versus interventional management, and key co-morbidities. The findings will be evaluated using the Grading of Recommendation Assessment, Development and Evaluation, informed by patient and public involvement work.

Ethics and dissemination

This is a systematic review of data in the public domain and does not require ethical approval. Dissemination will include presentations to key vascular and patient organisations, publication in a peer-reviewed journal, and an open-access repository of the study data.

Registration details

PROSPERO ID: CRD42023389262

For peer review only

For peer review only - http://bmjopen.bmj.com/site/about/guidelines.xhtml

Strengths and limitations of this study

1. This network meta-analysis (NMA) aims to address a critical knowledge gap in the clinical management of patients with peripheral arterial disease (PAD), as no existing or planned randomised controlled trials have directly compared the current most commonly recommended antithrombotic regimens.

2. The development of this protocol was informed by people with lived experience of PAD and plans further collaborative work with patients during the analysis and interpretation phases.

3. The NMA will comprehensively review important outcomes related to clinical effectiveness and adverse events for patients, while providing granularity through a series of planned subgroup analyses. Understanding potential differences between various antithrombotic combinations for key patient groups will provide clinicians and patients with important insights for each treatment option and may facilitate a more tailored approach to medical therapy.

4. The number of possible combinations of antithrombotic agents and doses is large and will require a pragmatic combining of these data. However, this review aims to provide a definitive synthesis of all evidence currently available on the topic, while patient involvement in the interpretation phase will ensure decisions leading to important conclusions are acceptable to patients.

5. Based on pre-existing knowledge of the field, the authors anticipate high heterogeneity between studies related to the inclusion/exclusion criteria of the available literature. Therefore, this protocol specifies robust, pre-specified sensitivity analyses to explore these.

For peer review only

For peer review only - http://bmjopen.bmj.com/site/about/guidelines.xhtml

Introduction

Peripheral arterial disease (PAD) describes a narrowing or occlusion of the peripheral arteries, which typically affects the lower limbs and is most often caused by atherosclerosis or atherothrombosis [1]. PAD may be asymptomatic or cause symptoms such as intermittent claudication (IC), where diminished circulation leads to pain in the lower limb on exertion that is relieved by rest [2]. More severe PAD can result in chronic limb-threatening ischaemia (CLTI), a clinical pattern representing threatened limb viability. CTLI is characterised by chronic, inadequate tissue perfusion at rest and is defined by ischaemic rest pain and/or tissue loss [3,4]. In the UK, around 2.2 million people have some degree of PAD, while the prevalence rises with age to 16% in those aged over 70 years [2,5–7]. Between 10 and 30% of individuals with PAD experience IC [3], while the population prevalence of reported CTLI is between 0.4 and 2% [3,4]. Other risk factors for both the development and progression of PAD include male gender, smoking, hypertension, diabetes, dyslipidaemia, and obesity [3,5].

The direct consequences of PAD include impaired quality of life [2,8], psychosocial sequelae [9], tissue loss (ulceration and gangrene) in CLTI [3], amputation [10], and procedural complications resulting from invasive treatments [2]. Patients with PAD are three times more likely to die of cardiovascular causes including myocardial infarction, stroke and suffer from vascular dementia, renovascular disease, and mesenteric disease [1,11,12]. Moreover, 10–15% of patients presenting with IC die of cardiovascular causes over the following five years, while 20% experience non-fatal cardiovascular events [8]. Therefore, guidelines recommend the assessment of cardiovascular risk, and management of all key modifiable risk factors such as smoking, glycaemic control, dyslipidaemia, hypertension, body weight, and exercise levels [3,13].

Antithrombotic pharmacological therapy, which may involve one or more antiplatelet and/or anticoagulant agents, has been repeatedly demonstrated in randomised controlled trials (RCTs) to reduce occlusive vascular events or their recurrence [14–22]. However, the optimal antithrombotic regimen for the management of PAD is contentious, both overall and in key sub-populations such as those with diabetes. Multiple RCTs compared the investigational product to aspirin monotherapy [14–16,19], which is not the recommended agent for the medical management of symptomatic PAD [3,23]. Furthermore, guidance from the National Institute for Health and Care Excellence (NICE) is incongruous; NICE Technology appraisal guidance 210 (TA210) states “Clopidogrel is recommended as an option to prevent occlusive vascular events for people who have … PAD or multivascular disease” [23], while NICE Technology appraisal guidance 607 (TA607) states “Rivaroxaban plus aspirin is recommended … as an option for preventing atherothrombotic events in adults with coronary artery disease or symptomatic PAD who are at high risk of ischaemic events” [24]. Antithrombotic therapy for patients undergoing interventions such as surgical bypass or endovascular intervention have separate recommendations.

For peer review only - http://bmjopen.bmj.com/site/about/guidelines.xhtml

The current guidance on the medical management of PAD generates confusion for both clinicians and patients, while variation in clinical practice may cause harm. No published RCT has directly compared the two NICE recommended antithrombotic regimens, clopidogrel monotherapy and rivaroxaban plus aspirin, in patients with PAD. Furthermore, there is no RCT comparison of these antithrombotic medications in patients with PAD either underway or being planned. Therefore, this network meta-analysis (NMA) aims to address a critical unanswered question in daily clinical practice, and understand the relative efficacy and risks associated with each antithrombotic regimen in preventing cardiovascular events, limb loss, death, and key adverse events in different patient groups with PAD.

Aims

To understand the relative efficacy and safety of previously investigated antithrombotic medications in preventing major cardiovascular events, limb loss, mortality and bleeding events in patients with symptomatic lower limb PAD. A secondary aim is to investigate whether different patient groups have superior outcomes from different antithrombotic regimens.

For peer review only

For peer review only - http://bmjopen.bmj.com/site/about/guidelines.xhtml

Method

The full protocol is available online [25], but in summary a systematic review and network meta-analysis of antithrombotic agents for patients with PAD will be undertaken. The review process will run from November 2022, to October 2023. The review is registered with the PROSPERO database [26], and will be reported in line with the latest PRISMA guidelines and relevant extensions for search strategies and NMAs [27–29].

Types of studies

All published and available unpublished RCT trial results will be included. All other study designs, including cross-over study designs and non-randomised studies, will be excluded.

Types of participants

People with PAD will be eligible, defined as (1) symptoms and a diagnosis of PAD by a clinician with experience in PAD and/or (2) a previous procedure to treat PAD (re-vascularisation procedure or amputation) and/or (3) objective evidence of lower limb arterial malperfusion. The ankle brachial pressure index (ABPI) is the most common objective measure of lower limb perfusion. Differing definitions of PAD exist using ABPI; for the avoidance of doubt an ABPI of ≤0.9 will be used as objective evidence of malperfusion.
No limits will be set on age, country, or previous therapy.

It is known that trials relevant to this review included patients with a variety of atherosclerotic disease phenotypes (e.g., coronary heart disease and/or ischaemic stroke in addition to PAD). Additionally, some trials are known to have included patients with carotid artery atherosclerotic disease within the PAD subgroup. These will be included provided that (1) a defined set of patients with symptomatic PAD was included, (2) one or more outcomes was specifically reported for the PAD subgroup, and (3) patients without lower limb PAD do not comprise greater than 25% of the PAD subgroup, but do have some other form of atherosclerotic pathology.

Types of interventions

Trials comparing one antithrombotic regimen to another, or to placebo, will be eligible for inclusion. Antithrombotic medication regimen will be defined as any individual or combination of medications listed in the British National Formulary as an antiplatelet, anticoagulant, glycoprotein IIb/IIIa inhibitor (Supplemental material) and/or any other individual or combination of medications reporting to inhibit platelets or fibrin aggregation in thrombus formation [30]. No restriction will be placed on dose or route of administration. However, studies which combine medication regimens with non-medication-based

For peer review only

For peer review only - http://bmjopen.bmj.com/site/about/guidelines.xhtml

co-interventions in a single arm will be excluded. Combinations of antithrombotic agents (for example clopidogrel with aspirin) will be analysed as separate groups to the individual agents.

Patient and public involvement (PPI)

This research proposal was developed with assistance from the local NIHR Research Design Service (RDS) and was informed by Patient and Public Involvement (PPI) work with a focus group of people with lived experience of PAD [31]. Themes emerging from the PPI group included surprise about the level of cardiovascular risk for patients with PAD and shock at the discordance in current guidelines. The PPI work highlighted that people with PAD most valued being alive without disabling complications such as stroke, heart attack or amputation, and felt that further research was important to establish the best antithrombotic agent/combination and if possible offer tailored advice for key patient groups.

Planned further PPI

Following data extraction, a visual representation of the network for each outcome will be created. This, along with the composite outcomes from studies that are being combined, risk of bias and difference in patient demographics of each included study will be discussed at a PPI focus group. This will ascertain their perception about the quality of evidence included in the study, which by extension may inform decision-making about treatment options recommended to patients. This will occur prior to the analyses being run, thereby avoiding any potential bias from the outcomes of the NMA.

The results of the analysis are likely to be numerous and complex in their nature. A second PPI focus group will discuss the outcomes of the analysis and distil the results down to a meaningful level for patients and members of the public to interpret. This is intended to facilitate dissemination and ensure that patients with PAD can access the information to make an informed decision about the best management for them with their doctor. A summary of these PPI findings will be reported jointly with the analysis.

Search methods

An information specialist developed a comprehensive literature search in line with the guidelines laid out in the Cochrane handbook, aiming for a high level of sensitivity [32]. The search strategy underwent peer review by the Cochrane Vascular information specialist, prior to being run [32]. Searches will be performed, in English only, on Medline, Embase, Cochrane (CENTRAL), Web of Science and Google Scholar. Searches for ongoing trials will be repeated via the World Health Organisation International Clinical Trials Registry platform search portal, with supplemental searches as recommended in the Cochrane handbook [31]. All databases will be searched from their inception. No limits will be placed on study language, although a study will be excluded if a translation is not available or feasible to arrange. The reference lists of included studies will be hand searched for further articles. All searches

For peer review only - http://bmjopen.bmj.com/site/about/guidelines.xhtml

will be repeated prior to the end of the meta-analysis to identify any subsequently published literature before the dataset is locked.

Selection of studies

The output of all searches will be imported into Covidence systematic review management software and screened for duplicates [33]. Once duplicates have been removed, two team members will independently screen the list of original papers by title and abstract to identify all RCTs of antithrombotic medication reporting one of the primary outcomes. For identified studies, all additional data sources will be sought utilising focussed searches for further published articles, published letters, trial registry entries, and requests for clinical study reports submitted to medical regulatory authorities. These documents will be bundled and screened in totality against the eligibility criteria independently by two blinded reviewers. The reviewers will attempt to resolve any disagreements once unblinded and a third reviewer will act as a tie breaker where no consensus can be reached.

Data extraction and management

Two team members will extract the data from the full text and supplementary materials of included studies into a pre-piloted data collection form in Covidence developed in collaboration with the statistician. Data on RCT design, participant baseline characteristics, study interventions, methods, all reported study outcomes, results, and the authors' conclusions will be extracted and recorded as detailed in the Cochrane handbook.

Where PAD patients represent a subgroup of the overall study population, data will be collected for both the study population as a whole and the PAD patient subgroup where available. Details of the PAD subgroup including disease status and proportion of non-lower limb atherosclerotic patients will also be recorded. This will permit a sensitivity analysis of the likelihood that using whole-study data rather than subgroup-specific data would introduce bias within the analysis of adverse events (AEs). Where multiple timepoints in follow-up are reported, the longest timepoint shall primarily be used for all outcomes, with subsequent sensitivity analyses exploring the effect of the timepoint of outcome measurement.

Objectives

Primary objectives

1. Define the relative efficacy and hierarchy of efficacy of all antithrombotic medication regimens, previously investigated in RCTs, at reducing the risk of major adverse cardiovascular events (MACE), in patients with PAD.

For peer review only - http://bmjopen.bmj.com/site/about/guidelines.xhtml

2. Define the relative efficacy and hierarchy of efficacy of all antithrombotic medication regimens, previously investigated in RCTs, at reducing the risk of major adverse limb-related events (MALE), in patients with PAD.
3. Define the relative efficacy and hierarchy of efficacy of all antithrombotic medication regimens, previously investigated in RCTs, at reducing the risk of death from any cause, in patients with PAD.
4. Define the relative risk and hierarchy of risk of all antithrombotic medication regimens, previously investigated in RCTs, of serious adverse events (AEs) including fatal bleeding, gastrointestinal bleeding, intracranial bleeding, severe bleeding into any major organ, bleeding requiring blood transfusion and/or return to theatre, and bleeding requiring admission to hospital, in patients with PAD.

Secondary objectives

5. Define the relative risk and hierarchy of risk of all antithrombotic medication regimens, previously investigated in RCTs, of any other recorded adverse drug effects, in patients with PAD.
6. Define the relative compliance to differing antithrombotic medication regimens, previously investigated in RCTs, in patients with PAD.
7. Explore the available data for differential efficacy and/or risks of all antithrombotic medication regimens (objectives 1-4), previously investigated in RCTs, in different subgroups including by gender, age, ethnicity, disease status (asymptomatic/IC/CLTI), conservative versus interventional management, type of interventional management, co-morbidities (other disease states), in patients with PAD.
8. Explore whether any other subgroups of patient or disease characteristics are sufficiently well reported in the included RCTs to establish an analysis and, if so, conduct that subgroup analysis.
9. Establish whether the teams of RCTs that form the judgement forming segments of the networks for the primary objectives are willing to collaborate and have sufficient data to undertake future individual patient network meta-analysis.

Outcomes

Co-primary outcomes:

Co-primary efficacy outcome 1: composite of major adverse cardiovascular events, as defined in available literature, but to include acute coronary syndrome, ischaemic stroke, and cardiovascular death.

For peer review only - http://bmjopen.bmj.com/site/about/guidelines.xhtml

Co-primary efficacy outcome 2: composite of major adverse limb-related events, as defined in available literature, but to include acute limb ischaemia (and embolectomy/thrombectomy/thrombolysis), major amputation (at or above ankle), or need for peripheral revascularisation.

Co-primary efficacy outcome 3: all-cause mortality.

Co-primary safety outcome 4: major bleeding, as defined in available literature, but to include fatal bleeding, symptomatic bleeding in a critical area or organ, such as intracranial, intraspinal, intraocular, retroperitoneal, intra-articular or pericardial, or intramuscular with compartment syndrome, bleeding causing a fall in haemoglobin level of 20 g/L or more, and/or bleeding requiring transfusion of red cells or whole blood.

Secondary outcomes:

Secondary efficacy outcomes will include individual components of MACE and MALE outcomes; cardiovascular death, acute coronary syndrome, ischaemic stroke, major amputation, acute limb ischaemia, thrombectomy/thrombolysis, and need for a subsequent revascularisation procedure.

Secondary safety outcomes will include individual outcomes of fatal bleeding, gastrointestinal bleeding, intracranial bleeding, severe bleeding into any major organ, bleeding requiring blood transfusion and/or return to theatre, and bleeding requiring admission to hospital, venous thromboembolism, rash, discontinuation of assigned therapy for any reason, gastrointestinal symptoms resulting in discontinuation of assigned therapy. Additionally, any further drug-related AEs reported will be included to allow for the identification of unexpected AEs. Adherence to therapy by any quantitative measure will be included, where reported.

Risk of bias assessment

Two study members with domain and meta-analysis expertise will independently assess all included trials for risk of bias using the “ROB 2” tool [34], then areas of uncertainty in these assessments will look to be resolved by reference to collateral information sources as described above (trial registries and regulatory submissions) and contact with the primary investigators if necessary. A risk of bias assessment will be undertaken separately for primary outcome analyses and subgroup analyses.

Measures of treatment effects

For all binary outcomes, the preferred data to collect will be absolute numbers of events and numbers at risk, and risk ratios will be calculated. If the absolute number of events is not available, risk or odds ratios with defined confidence intervals and/or standard error will be the preferred alternative. For continuous outcomes, the mean and standard deviation of the group will be the first choice of measure.

For peer review only - http://bmjopen.bmj.com/site/about/guidelines.xhtml

Unit of analysis issues

Analyses will be conducted at medication regimen level. Dose will not be primarily considered but a secondary sensitivity analysis on the primary outcomes will be undertaken to see if any dose effects are evident. It is not anticipated that any trials will report data in another manner regarding this, however advice will be sought from the statistician should this occur.

Missing data

Only published data will be analysed. Missing data will be considered within the risk of bias assessment. If data is made available through direct correspondence it will be published as an appendix to the review and in the studies data repository.

Transitivity

Recruitment criteria differ between the RCTs and as such the transitivity assumption may be threatened. This is perceived to particularly be the case when considering trials of stable PAD compared with trials of peri-/post-procedural PAD. The validity of the transitivity assumption will be assessed quantitatively by considering the incoherence factor, which involves the comparison of direct and indirect effective estimates for each pair-wise comparison in the network, where they both exist); this will be evaluated using both the local and global strategies detailed in the Cochrane handbook [31].

Assessment of reporting

Aspirin is expected to be the most common comparator within RCTs in this NMA, which will facilitate exploration of publication bias using comparison-adjusted funnel plots [35]. The team has considered selective reporting within trials throughout the study design phase and will qualitatively consider the effect of this during subsequent phases.

Data synthesis

The full analysis plan is available online [36]. Studies will be summarised including the direct comparisons made, population characteristics, and characteristics of those patients with PAD. The medication regimen for both overall and PAD populations will be summarised separately.

Antithrombotic regimens will be grouped into common nodes based on the drugs used in that arm. A network diagram will be constructed for each outcome, where the size of each node is proportional to the number of patients assigned to that intervention, and the thickness of each line is based on the

For peer review only - http://bmjopen.bmj.com/site/about/guidelines.xhtml

inverse of the variance of the direct comparison. Interventions that are absent from a particular network will be highlighted.

Analysis of primary and secondary outcomes will be undertaken on a whole network basis, subject to the checks of assumptions outlined above, wherever networks can be formed based on published data. All NMAs will create pairwise comparisons of medication regimens, and a ranking of all medication regimens will be produced with risk of bias estimates published alongside. Subgroup analyses are discussed further below.

We anticipate a complex NMA based upon prior knowledge. The analysis is planned by the statistician (CH) in R Statistical Software (v4.1) [37], primarily using the *netmeta* package [38]. If the transitivity assumption is violated as the result of differing RCT recruitment criteria, then separate networks will be established to allow meaningful analysis of these data.

Subgroup analysis

The PPI group highlighted the importance of being able to provide individualised recommendations to patients. Therefore, the intent is to extract all published results for subgroups and perform all NMAs that are possible. Anticipated subgroups are PAD disease state (such as IC & CLTI), sex, age, ethnicity, key co-morbidities, and peri-procedural status. We also intend to analyse any other subgroups that have sufficient data published but are not anticipated.

Subgroup analyses as described above, will be undertaken wherever sub-networks can be formed based on published data. The inclusion of these sub-analyses will likely be susceptible to non-reporting bias as they are less likely to have formed part of the original RCT per-protocol analysis plan. Candidate interventions that are absent due to non-publication of a particular subgroup analysis will be clearly identified.

Sensitivity analysis

Pre-specified sensitivity analyses, as permitted by available data, will explore consistency in the findings regarding (1) patients with stable versus periprocedural PAD, (2) primary outcomes limited to patients with PAD only (to ascertain whether adverse event rates in trials of mixed atherosclerotic disease e.g., coronary heart disease and stroke, as well as PAD, are applicable to the PAD only population, (3) inclusion of studies at low risk of bias only, (4) restriction of included patients to those with IC and/or CTLI in isolation, (5) primary outcomes by dose of antithrombotic agents (variable dosing strategies, particularly of aspirin, are anticipated to affect adverse event rates), and (6) shorter and longer timepoints (as the optimal antithrombotic regimen may change over time for patients with PAD). Further post-hoc sensitivity analyses may be developed and will be reported transparently as post-hoc in the final report.

For peer review only - http://bmjopen.bmj.com/site/about/guidelines.xhtml

Confidence in cumulative evidence

A summary of findings table will be constructed for the key outcomes, including MACE, MALE, all-cause mortality, major amputation as an individualised outcome, and major bleeding. The underlying quality of evidence for each of these outcomes will be assessed according to the GRADE framework for NMAs, which classifies interventions by both the relative treatment effect size and certainty of evidence [39,40].

Ethics and dissemination

Clinical Impact

The results will be presented to clinical domain specialists at UK national and international vascular and endovascular conferences. Presentations will be made to key stakeholders including NICE, vascular and endovascular societies/charities, and the all-party parliamentary group for vascular and venous disease. The lead applicants' institutions public relations team will maximise the visibility and availability of the results, both to patients and clinicians, through social and traditional media. The results will be disseminated through the UK national societies of vascular specialists and general practitioners to maximise the impact on clinical practice. The authors anticipate that the results of the research would be sufficient to trigger a review of the current NICE guidelines.

Patient Impact

The patient accessible report co developed with the PPI group will be submitted to UK vascular disease charities, such as the Circulation Foundation and the British Heart Foundation, for publication on their websites.

Data storage

The data generated from this research will be an essential output. The data collected from screening, risk assessments, study results, and statistical coding will be made available on the lead applicants institution research data repository to ensure ongoing future access. This will facilitate research efficiency if/when future trials become available, and the analysis requires updating. We will seek to establish a resource that can be added-to, re-evaluated, or re-analysed with future trail data. A link to the data repository will be provided in the PROSPERO record for the systematic review.

Publications

The authors anticipate that the primary paper will be published, open access, in a high impact cardiovascular journal. Any further publications will also be submitted, open access.

For peer review only - http://bmjopen.bmj.com/site/about/guidelines.xhtml

Acknowledgements

The authors thank Candida Fenton from the Cochrane Vascular Group, University of Edinburgh, Edinburgh, United Kingdom, for peer review of the search strategy.

Funding:

This project is funded by the National Institute for Health and Care Research (NIHR) under its Research for Patient Benefit (RfPB) Programme (Grant Reference Number NIHR204123). The views expressed are those of the author(s) and not necessarily those of the NIHR or the Department of Health and Social Care.

Competing interest statement: We have read and understood the BMJ Group policy on declaration of interests and declare the following interests: Nil - Except above funding.

Contributor statement: All authors drafted and revised the manuscript. Mr Iain N Roy is the guarantor for this work. The corresponding author attests that all listed authors meet authorship criteria and that no others meeting the criteria have been omitted.

For peer review only

For peer review only - http://bmjopen.bmj.com/site/about/guidelines.xhtml

References

1 Morley RL, Sharma A, Horsch AD, *et al.* Peripheral artery disease. *BMJ* 2018;**360**:j5842. doi:10.1136/bmj.j5842

2 Conte MS, Pomposelli FB, Clair DG, *et al.* Society for Vascular Surgery practice guidelines for atherosclerotic occlusive disease of the lower extremities: Management of asymptomatic disease and claudication. *J Vasc Surg* 2015;**61**:2S-41S.e1. doi:10.1016/j.jvs.2014.12.009

3 Aboyans V, Ricco JB, Bartelink MLEL, *et al.* 2017 ESC Guidelines on the Diagnosis and Treatment of Peripheral Arterial Diseases, in collaboration with the European Society for Vascular Surgery (ESVS). *Eur Heart J* 2018;**39**:763–816. doi:10.1093/eurheartj/ehx095

4 Farber A. Chronic Limb-Threatening Ischemia. *New England Journal of Medicine* 2018;**379**:171–80. doi:10.1056/nejmcp1709326

5 Song P, Rudan D, Zhu Y, *et al.* Global, regional, and national prevalence and risk factors for peripheral artery disease in 2015: an updated systematic review and analysis. *Lancet Glob Health* 2019;**7**:e1020–30. doi:10.1016/S2214-109X(19)30255-4

6 Criqui MH, Aboyans V. Epidemiology of Peripheral Artery Disease. *Circ Res* 2015;**116**:1509–26. doi:10.1161/CIRCRESAHA.116.303849

7 Institute for Health Metrics and Evaluation (IHME). Global Burden of Disease. 2019.https://ghdx.healthdata.org (accessed 8 Apr 2022).

8 National Clinical Guideline Centre (UK). *Lower Limb Peripheral Arterial Disease: Diagnosis and Management [Internet]*. London: Royal College of Physicians (UK): : National Institute for Health and Clinical Excellence: Guidance 2012. https://www.ncbi.nlm.nih.gov/books/NBK299071/

9 Shishehbor MH, White CJ, Gray BH, *et al.* Critical Limb Ischemia. *J Am Coll Cardiol* 2016;**68**:2002–15. doi:10.1016/j.jacc.2016.04.071

10 Gerhard-Herman MD, Gornik HL, Barrett C, *et al.* 2016 AHA/ACC Guideline on the Management of Patients With Lower Extremity Peripheral Artery Disease: Executive Summary: A Report of the American College of Cardiology/American Heart Association Task Force on Clinical Practice Guidelines. *J Am Coll Cardiol* 2017;**69**:1465–508. doi:10.1016/j.jacc.2016.11.008

11 Cea-Soriano L, Fowkes FGR, Johansson S, *et al.* Time trends in peripheral artery disease incidence, prevalence and secondary preventive therapy: A cohort study in the Health Improvement Network in the UK. *BMJ Open* 2018;**8**:1–7. doi:10.1136/bmjopen-2017-018184

12 Foley TR, Armstrong EJ, Waldo SW. Contemporary evaluation and management of lower extremity peripheral artery disease. *Heart* 2016;**102**:1436–41. doi:10.1136/HEARTJNL-2015-309076

13 National Institute for Health and Care Excellence. Peripheral arterial disease: diagnosis and management. Clinical guideline [CG147]. 2012.https://www.nice.org.uk/guidance/cg147 (accessed 20 Apr 2022).

For peer review only - http://bmjopen.bmj.com/site/about/guidelines.xhtml

14 Anand SS, Widimsky P, Alings M, *et al.* Rivaroxaban with or without aspirin in patients with stable peripheral or carotid artery disease: an international, randomised, double-blind, placebo-controlled trial. *The Lancet (British edition)* 2018;**391**:219–29. doi:10.1016/S0140-6736(17)32409-1

15 Eikelboom JW, Connolly SJ, Bosch J, *et al.* Rivaroxaban with or without aspirin in stable cardiovascular disease. *New England Journal of Medicine* 2017;**377**:1319–30. doi:10.1056/NEJMoa1709118

16 CAPRIE Steering Committee. A randomised, blinded, trial of clopidogrel versus aspirin in patients at risk of ischaemic events (CAPRIE). *Lancet* 1996;**348**:1329–39. doi:10.1016/s0140-6736(96)09457-3

17 Becquemin J-P. Effect of Ticlopidine on the Long-Term Patency of Saphenous-Vein Bypass Grafts in the Legs. *N Engl J Med* 1997;**337**:1726–31. doi:10.1056/NEJM199712113372404

18 Hiatt WR, Fowkes FGR, Heizer G, *et al.* Ticagrelor versus Clopidogrel in Symptomatic Peripheral Artery Disease. *N Engl J Med* 2017;**376**:32–40. doi:10.1056/NEJMoa1611688

19 Bonaca MP, Bauersachs RM, Anand SS, *et al.* Rivaroxaban in Peripheral Artery Disease after Revascularization. *N Engl J Med* 2020;**382**:1994–2004. doi:10.1056/NEJMoa2000052

20 Strobl FF, Brechtel K, Schmehl J, *et al.* Twelve-month results of a randomized trial comparing mono with dual antiplatelet therapy in endovascularly treated patients with peripheral artery disease. *J Endovasc Ther* 2013;**20**:699–706. doi:10.1583/13-4275MR.1

21 Burdess A, Nimmo AF, Garden OJ, *et al.* Randomized controlled trial of dual antiplatelet therapy in patients undergoing surgery for critical limb ischemia. *Ann Surg* 2010;**252**:37–42. doi:10.1097/SLA.0b013e3181e40dde

22 Warfarin Antiplatelet Vascular Evaluation Trial Investigators, Anand S, Yusuf S, *et al.* Oral anticoagulant and antiplatelet therapy and peripheral arterial disease. *N Engl J Med* 2007;**357**:217–27. doi:10.1056/NEJMoa065959

23 National Institute for Health and Care Excellence. Clopidogrel and modified-release dipyridamole for the prevention of occlusive vascular events. Technology appraisal guidance [TA210]. 2010.https://www.nice.org.uk/guidance/ta210 (accessed 20 Apr 2022).

24 National Institute for Health and Care Excellence. Rivaroxaban for preventing atherothrombotic events in people with coronary or peripheral artery disease. Technology appraisal guidance [TA607]. 2019.https://www.nice.org.uk/guidance/ta607 (accessed 20 Apr 2022).

25 Roy I, Sidebottom D. Protocol - Systematic review & network meta-analysis of randomised controlled trials comparing antithrombotic drugs for cardiovascular risk reduction in patients with lower limb peripheral arterial disease. 2022. doi:10.24376/rd.sgul.21591216.v1

26 Roy I, Sidebottom DB, Holt P, *et al.* Antithrombotic drugs for cardiovascular risk reduction in patients with lower limb peripheral arterial disease: protocol for a systematic review and network meta-analysis of randomised controlled trials. PROSPERO 2023. https://www.crd.york.ac.uk/prospero/display_record.php?ID=CRD42023389262 (accessed 5 Jan 2023).

For peer review only - http://bmjopen.bmj.com/site/about/guidelines.xhtml

27 Page MJ, McKenzie JE, Bossuyt PM, *et al.* The PRISMA 2020 statement: an updated guideline for reporting systematic reviews. *BMJ* 2021;**372**:n71. doi:10.1136/bmj.n71

28 Rethlefsen ML, Kirtley S, Waffenschmidt S, *et al.* PRISMA-S: an extension to the PRISMA Statement for Reporting Literature Searches in Systematic Reviews. *Syst Rev* 2021;**10**:39. doi:10.1186/s13643-020-01542-z

29 Hutton B, Salanti G, Caldwell DM, *et al.* The PRISMA extension statement for reporting of systematic reviews incorporating network meta-analyses of health care interventions: checklist and explanations. *Ann Intern Med* 2015;**162**:777–84. doi:10.7326/M14-2385

30 Joint Formulary Committee. British National Formulary (online). 2022.http://www.medicinescomplete.com (accessed 20 Apr 2022).

31 Higgins J, Thomas J, Chandler J, *et al.* Cochrane Handbook for Systematic Reviews of Interventions. Cochrane Handbook for Systematic Reviews of Interventions version 6.3 (updated Feburary 2022). 2022.https://training.cochrane.org/handbook (accessed 20 Apr 2022).

32 Roy I, Sidebottom DB, John-Pierre K. Search strategy - Systematic review & network meta-analysis of randomised controlled trials comparing antithrombotic drugs for cardiovascular risk reduction in patients with lower limb peripheral arterial disease. 2023. doi:10.24376/rd.sgul.21687866

33 Veritas Health Innovation. Covidence systematic review software. 2022.www.covidence.org (accessed 20 Apr 2022).

34 Sterne JAC, Savović J, Page MJ, *et al.* RoB 2: a revised tool for assessing risk of bias in randomised trials. *BMJ* 2019;**366**:l4898. doi:10.1136/bmj.l4898

35 Chaimani A, Salanti G. Using network meta-analysis to evaluate the existence of small-study effects in a network of interventions. *Res Synth Methods* 2012;**3**:161–76. doi:10.1002/jrsm.57

36 Roy I, Huang C. Analysis Plan - Systematic review & network meta-analysis of randomised controlled trials comparing antithrombotic drugs for cardiovascular risk reduction in patients with lower limb peripheral arterial disease. 2023. doi:10.24376/rd.sgul.21688040

37 R Core Team. R: A language and environment for statistical computing. 2022.

38 Rücker G, Krahn U, König J, *et al.* netmeta: Network Meta-Analysis using Frequentist Methods. 2022.https://cran.r-project.org/package=netmeta

39 Brignardello-Petersen R, Izcovich A, Rochwerg B, *et al.* GRADE approach to drawing conclusions from a network meta-analysis using a partially contextualised framework. *The BMJ* 2020;**371**. doi:10.1136/bmj.m3907

40 Brignardello-Petersen R, Florez ID, Izcovich A, *et al.* GRADE approach to drawing conclusions from a network meta-analysis using a minimally contextualised framework. *The BMJ* 2020;**371**:1–8. doi:10.1136/bmj.m3900

For peer review only - http://bmjopen.bmj.com/site/about/guidelines.xhtml

Supplemental Material

Antithrombotic medications currently named in the British National Formulary are aspirin, clopidogrel, dipyridamole, prasugrel, ticagrelor, cangrelor, warfarin sodium, acenocoumarol, phenindione, apixaban, edoxaban, rivaroxaban, dabigatran etexilate, heparin (unfractionated), dalteparin sodium, enoxaparin sodium, tinzaparin sodium, danaparoid sodium, argatroban monohydrate, bivalirudin, epoprostenol, and fondaparinux.ubmission

For peer review only

PRISMA-P 2015 Checklist

This checklist has been adapted for use with protocol submissions to *Systematic Reviews* from Table 3 in Moher D et al: Preferred reporting items for systematic review and meta-analysis protocols (PRISMA-P) 2015 statement. *Systematic Reviews* 2015 **4**:1

Section/topic	#	Checklist item	Information reported: Yes	Information reported: No	Line number(s)
**ADMINISTRATIVE INFORMATION**					
**Title**					
Identification	1a	Identify the report as a protocol of a systematic review	☒	☐	1:1-4
Update	1b	If the protocol is for an update of a previous systematic review, identify as such	☐	☒	N/A
**Registration**	2	If registered, provide the name of the registry (e.g., PROSPERO) and registration number in the Abstract	☒	☐	2:37
**Authors**					
Contact	3a	Provide name, institutional affiliation, and e-mail address of all protocol authors; provide physical mailing address of corresponding author	☒	☐	1:10-25
Contributions	3b	Describe contributions of protocol authors and identify the guarantor of the review	☒	☐	1:44-46
**Amendments**	4	If the protocol represents an amendment of a previously completed or published protocol, identify as such and list changes; otherwise, state plan for documenting important protocol amendments	☐	☒	N/A
**Support**					
Sources	5a	Indicate sources of financial or other support for the review	☒	☐	1:30-33
Sponsor	5b	Provide name for the review funder and/or sponsor	☒	☐	1:30-35
Role of sponsor/funder	5c	Describe roles of funder(s), sponsor(s), and/or institution(s), if any, in developing the protocol	☒	☐	1:37-39
**INTRODUCTION**					
**Rationale**	6	Describe the rationale for the review in the context of what is already known	☒	☐	4:1-47
**Objectives**	7	Provide an explicit statement of the question(s) the review will address with reference to participants, interventions, comparators, and outcomes (PICO)	☒	☐	4:51-54
**METHODS**					

BioMed Central The Open Access Publisher

For peer review only - http://bmjopen.bmj.com/site/about/guidelines.xhtml

Section/topic	#	Checklist item	Information reported Yes	Information reported No	Line number(s)
**Eligibility criteria**	8	Specify the study characteristics (e.g., PICO, study design, setting, time frame) and report characteristics (e.g., years considered, language, publication status) to be used as criteria for eligibility for the review	☒	☐	5:1-41
**Information sources**	9	Describe all intended information sources (e.g., electronic databases, contact with study authors, trial registers, or other grey literature sources) with planned dates of coverage	☒	☐	6:15-23
**Search strategy**	10	Present draft of search strategy to be used for at least one electronic database, including planned limits, such that it could be repeated	☒	☐	6:13 (cited)
STUDY RECORDS					
Data management	11a	Describe the mechanism(s) that will be used to manage records and data throughout the review	☒	☐	6:25-35
Selection process	11b	State the process that will be used for selecting studies (e.g., two independent reviewers) through each phase of the review (i.e., screening, eligibility, and inclusion in meta-analysis)	☒	☐	6:28-43
Data collection process	11c	Describe planned method of extracting data from reports (e.g., piloting forms, done independently, in duplicate), any processes for obtaining and confirming data from investigators	☒	☐	6:39-52
**Data items**	12	List and define all variables for which data will be sought (e.g., PICO items, funding sources), any pre-planned data assumptions and simplifications	☒	☐	6:41-52
**Outcomes and prioritization**	13	List and define all outcomes for which data will be sought, including prioritization of main and additional outcomes, with rationale	☒	☐	7:33-8:11
**Risk of bias in individual studies**	14	Describe anticipated methods for assessing risk of bias of individual studies, including whether this will be done at the outcome or study level, or both; state how this information will be used in data synthesis	☒	☐	8:13-19
DATA					
**Synthesis**	15a	Describe criteria under which study data will be quantitatively synthesized	☒	☐	9:1 (cited), 11-20
	15b	If data are appropriate for quantitative synthesis, describe planned summary measures, methods of handling data, and methods of combining data from studies, including any planned exploration of consistency (e.g., I^2, Kendall's tau)	☒	☐	8:41-48, 9:1 (cited) - 20
	15c	Describe any proposed additional analyses (e.g., sensitivity or subgroup analyses, meta-regression)	☒	☐	9:22-34
	15d	If quantitative synthesis is not appropriate, describe the type of summary planned	☒	☐	9:18-20
**Meta-bias(es)**	16	Specify any planned assessment of meta-bias(es) (e.g., publication bias across studies, selective reporting within studies)	☒	☐	8:50-55
**Confidence in cumulative evidence**	17	Describe how the strength of the body of evidence will be assessed (e.g., GRADE)	☒	☐	9:49-54

BioMed Central The Open Access Publisher

For peer review only - http://bmjopen.bmj.com/site/about/guidelines.xhtml

BMJ Open

Antithrombotic drugs for cardiovascular risk reduction in patients with lower limb peripheral arterial disease: protocol for a systematic review & network meta-analysis of randomised controlled trials

Journal:	*BMJ Open*
Manuscript ID	bmjopen-2023-072355.R1
Article Type:	Protocol
Date Submitted by the Author:	11-Jun-2023
Complete List of Authors:	Sidebottom, David; St George's University of London, Molecular and Clinical Sciences Research Institute; St George's University Hospitals NHS Foundation Trust, St Georges Vascular Institute<br>Huang, Chao; Hull York Medical School<br>Carradice, Daniel; Hull York Medical School; Hull University Teaching Hospitals NHS Trust<br>Holt, Peter; St George's University of London, Molecular and Clinical Sciences Research Institute; St George's University Hospitals NHS Foundation Trust, St George's Vascular Institute<br>John-Pierre, Karen; St George's University of London, NHS Liaison Librarian<br>Roy, Iain; St George's University of London, Molecular and Clinical Sciences Research Institute; St George's University Hospitals NHS Foundation Trust, St George's Vascular Institute
**Primary Subject Heading**:	Cardiovascular medicine
Secondary Subject Heading:	Evidence based practice
Keywords:	Anticoagulation < HAEMATOLOGY, Vascular medicine < INTERNAL MEDICINE, Vascular surgery < SURGERY, VASCULAR SURGERY, VASCULAR MEDICINE

SCHOLARONE™
Manuscripts

Protocol for a network meta-analysis

Antithrombotic drugs for cardiovascular risk reduction in patients with lower limb peripheral arterial disease: protocol for a systematic review & network meta-analysis of randomised controlled trials

Short title: Protocol for a NMA of antithrombotics for PAD

David B. Sidebottom *Academic Foundation Doctor* [1 2]
Chao Huang *Senior Lecturer in Statistics* [3]
Daniel Carradice *Senior Lecturer in Vascular and Endovascular Surgery* [3 4]
Peter Holt *Professor of Vascular Surgery* [1 2]
Karen John-Pierre *NHS Liaison Librarian* [1]
Iain N Roy *Hon. Senior lecturer in Vascular Surgery* [1 2*]

Affiliations

[1] St George's, University of London, London, United Kingdom

[2] St George's University Hospitals NHS Foundation Trust, London, United Kingdom

[3] Hull York Medical School, United Kingdom

[4] Hull University Teaching Hospitals NHS Trust, United Kingdom

[*]Address for correspondence

Mr Iain N Roy, Molecular and Clinical Sciences Research Institute, St. George's, University of London

E-mail: iroy@sgul.ac.uk

Word count: 3595 / 4000

Sponsor: St George's, University of London.

Role of the funder/sponsor: The funder peer reviewed but did not have any other role in the design, preparation, or writing of this protocol. The authors had full autonomy in all aspects of protocol development.

For peer review only

For peer review only - http://bmjopen.bmj.com/site/about/guidelines.xhtml

Copyright statement: This research was funded in whole, or in part, by NIHR (NIHR204123). For the purpose of open access, the author has applied a Creative Commons Attribution (CC BY) licence to any Author Accepted Manuscript version arising from this submission.'

For peer review only

For peer review only - http://bmjopen.bmj.com/site/about/guidelines.xhtml

Abstract [297 / 300 words]

Introduction

The optimal antithrombotic regimen to reduce the risk of vascular events in patients with peripheral arterial disease (PAD) is contentious. This systematic review and network meta-analysis aims to define the relative efficacy and risks of previously investigated antithrombotic medication regimens in preventing major cardiovascular events, vascular limb events, and mortality in patients with PAD.

Methods and analysis

A peer-reviewed, systematic search will be executed in English on Medline, Embase, Cochrane (CENTRAL), Web of Science, and Google Scholar databases in late 2022. The World Health Organisation International Clinical Trials Registry platform will also be searched for ongoing trials. Abstracts will be screened independently by two researchers for randomised controlled trials meeting the review criteria. All associated publications including the study protocol will be sought and evaluated together against pre-specified inclusion/exclusion criteria. Two researchers will extract the data into a pre-piloted extraction form. Risk of bias assessments will be performed using the Cochrane "Risk of Bias 2" criteria by individuals with domain expertise. All differences will be resolved by consensus or a third individual for ties.

Included trials will be summarised. A network meta-analysis will be performed, subject to checks of assumptions. Both primary and secondary outcomes will be analysed on a whole network basis. Pairwise comparisons and league tables will be produced. Pre-specified subgroup analyses will include sex, ethnicity, disease status, conservative versus interventional management, and key co-morbidities. The findings will be evaluated using the Grading of Recommendation Assessment, Development and Evaluation, informed by patient and public involvement work.

Ethics and dissemination

This is a systematic review of data in the public domain and does not require ethical approval. Dissemination will include presentations to key vascular and patient organisations, publication in a peer-reviewed journal, and an open-access repository of the study data.

Registration details

PROSPERO ID: CRD42023389262

For peer review only

For peer review only - http://bmjopen.bmj.com/site/about/guidelines.xhtml

Strengths and limitations of this study

1. A broad highly sensitive search strategy has been designed in collaboration with an experienced librarian to facilitate the most comprehensive review of this topic area in existence.

2. This study and its aims were closely informed by people with lived experience of PAD, with further collaborative work with patients planned during the analysis and interpretation phases.

3. A comprehensive series of subgroup analyses will provide the granularity necessary to understand potential differences between various antithrombotic combinations for key patient groups.

4. The number of possible combinations of antithrombotic agents and doses is large and will require a pragmatic combining of these data.

5. A number of robust sensitivity analyses are pre-specified to explore anticipated high heterogeneity between studies, related to the inclusion/exclusion criteria of the available literature.

For peer review only - http://bmjopen.bmj.com/site/about/guidelines.xhtml

Introduction

Peripheral arterial disease (PAD) describes a narrowing or occlusion of the peripheral arteries, which typically affects the lower limbs and is most often caused by atherosclerosis or atherothrombosis [1]. PAD may be asymptomatic or cause symptoms such as intermittent claudication (IC), where diminished circulation leads to pain in the lower limb on exertion that is relieved by rest [2]. More severe PAD can result in chronic limb-threatening ischaemia (CLTI), a clinical pattern representing threatened limb viability. CTLI is characterised by chronic, inadequate tissue perfusion at rest and is defined by ischaemic rest pain and/or tissue loss [3,4]. In the UK, around 2.2 million people have some degree of PAD, while the prevalence rises with age to 16% in those aged over 70 years [2,5–7]. Between 10 and 30% of individuals with PAD experience IC [3], while the population prevalence of reported CTLI is between 0.4 and 2% [3,4]. Other risk factors for both the development and progression of PAD include male gender, smoking, hypertension, diabetes, dyslipidaemia, and obesity [3,5].

The direct consequences of PAD include impaired quality of life [2,8], psychosocial sequelae [9], tissue loss (ulceration and gangrene) in CLTI [3], amputation [10], and procedural complications resulting from invasive treatments [2]. Patients with PAD are three times more likely to die of cardiovascular causes including myocardial infarction, stroke and suffer from vascular dementia, renovascular disease, and mesenteric disease [1,11,12]. Moreover, 10–15% of patients presenting with IC die of cardiovascular causes over the following five years, while 20% experience non-fatal cardiovascular events [8]. Therefore, guidelines recommend the assessment of cardiovascular risk, and management of all key modifiable risk factors such as smoking, glycaemic control, dyslipidaemia, hypertension, body weight, and exercise levels [3,13].

Antithrombotic pharmacological therapy, which may involve one or more antiplatelet and/or anticoagulant agents, has been repeatedly demonstrated in randomised controlled trials (RCTs) to reduce occlusive vascular events or their recurrence [14–22]. However, the optimal antithrombotic regimen for the management of PAD is contentious, both overall and in key sub-populations such as those with diabetes. Multiple RCTs compared the investigational product to aspirin monotherapy [14–16,19], which is not the recommended agent for the medical management of symptomatic PAD [3,23]. Furthermore, guidance from the National Institute for Health and Care Excellence (NICE) is incongruous; NICE Technology appraisal guidance 210 (TA210) states “Clopidogrel is recommended as an option to prevent occlusive vascular events for people who have … PAD or multivascular disease” [23], while NICE Technology appraisal guidance 607 (TA607) states “Rivaroxaban plus aspirin is recommended … as an option for preventing atherothrombotic events in adults with coronary artery disease or symptomatic PAD who are at high risk of ischaemic events” [24]. Antithrombotic therapy for patients undergoing interventions such as surgical bypass or endovascular intervention have separate recommendations.

For peer review only - http://bmjopen.bmj.com/site/about/guidelines.xhtml

The current guidance on the medical management of PAD generates confusion for both clinicians and patients, while variation in clinical practice may cause harm. No published RCT has directly compared the two NICE recommended antithrombotic regimens, clopidogrel monotherapy and rivaroxaban plus aspirin, in patients with PAD. Furthermore, there is no RCT comparison of these antithrombotic medications in patients with PAD either underway or being planned. Therefore, this network meta-analysis (NMA) aims to address a critical unanswered question in daily clinical practice, and understand the relative efficacy and risks associated with each antithrombotic regimen in preventing cardiovascular events, limb loss, death, and key adverse events in different patient groups with PAD.

Aims

To understand the relative efficacy and safety of previously investigated antithrombotic medications in preventing major cardiovascular events, limb loss, mortality and bleeding events in patients with symptomatic lower limb PAD. A secondary aim is to investigate whether different patient groups have superior outcomes from different antithrombotic regimens.

For peer review only

For peer review only - http://bmjopen.bmj.com/site/about/guidelines.xhtml

Method

The full protocol is available online [25], but in summary a systematic review and network meta-analysis of antithrombotic agents for patients with PAD will be undertaken. The review process will run from November 2022, to October 2023. The review is registered with the PROSPERO database [26], and will be reported in line with the latest PRISMA guidelines and relevant extensions for search strategies and NMAs [27–29].

Types of studies

All published and available unpublished RCT trial results will be included. All other study designs, including cross-over study designs and non-randomised studies, will be excluded.

Types of participants

People with PAD will be eligible, defined as (1) symptoms and a diagnosis of PAD by a clinician with experience in PAD and/or (2) a previous procedure to treat PAD (re-vascularisation procedure or amputation) and/or (3) objective evidence of lower limb arterial malperfusion. The ankle brachial pressure index (ABPI) is the most common objective measure of lower limb perfusion. Differing definitions of PAD exist using ABPI; for the avoidance of doubt an ABPI of ≤0.9 will be used as objective evidence of malperfusion.
No limits will be set on age, country, or previous therapy.

It is known that trials relevant to this review included patients with a variety of atherosclerotic disease phenotypes (e.g., coronary heart disease and/or ischaemic stroke in addition to PAD). Additionally, some trials are known to have included patients with carotid artery atherosclerotic disease within the PAD subgroup. These will be included provided that (1) a defined set of patients with symptomatic PAD was included, (2) one or more outcomes was specifically reported for the PAD subgroup, and (3) patients without lower limb PAD do not comprise greater than 25% of the PAD subgroup but do have some other form of atherosclerotic pathology.

Types of interventions

Trials comparing one antithrombotic regimen to another, or to placebo, will be eligible for inclusion. Antithrombotic medication regimen will be defined as any individual or combination of medications listed in the British National Formulary as an antiplatelet, anticoagulant, glycoprotein IIb/IIIa inhibitor (Supplemental material) and/or any other individual or combination of medications reporting to inhibit platelets or fibrin aggregation in thrombus formation [30]. No restriction will be placed on dose or route of administration. However, studies which combine medication regimens with non-medication-based

For peer review only - http://bmjopen.bmj.com/site/about/guidelines.xhtml

co-interventions in a single arm will be excluded. Combinations of antithrombotic agents (for example clopidogrel with aspirin) will be analysed as separate groups to the individual agents.

Patient and public involvement (PPI)

This research proposal was developed with assistance from the local NIHR Research Design Service (RDS) and was informed by Patient and Public Involvement (PPI) work with a focus group of people with lived experience of PAD [31]. Themes emerging from the PPI group included surprise about the level of cardiovascular risk for patients with PAD and shock at the discordance in current guidelines. The PPI work highlighted that people with PAD most valued being alive without disabling complications such as stroke, heart attack or amputation, and felt that further research was important to establish the best antithrombotic agent/combination and if possible offer tailored advice for key patient groups.

Planned further PPI

Following data extraction, a visual representation of the network for each outcome will be created. This, along with the composite outcomes from studies that are being combined, risk of bias and difference in patient demographics of each included study will be discussed at a PPI focus group. This will ascertain their perception about the quality of evidence included in the study, which by extension may inform decision-making about treatment options recommended to patients. This will occur prior to the analyses being run, thereby avoiding any potential bias from the outcomes of the NMA.

The results of the analysis are likely to be numerous and complex in their nature. A second PPI focus group will discuss the outcomes of the analysis and distil the results down to a meaningful level for patients and members of the public to interpret. This is intended to facilitate dissemination and ensure that patients with PAD can access the information to make an informed decision about the best management for them with their doctor. A summary of these PPI findings will be reported jointly with the analysis.

Search methods

An information specialist developed a comprehensive literature search in line with the guidelines laid out in the Cochrane handbook, aiming for a high level of sensitivity [32]. The search strategy underwent peer review by the Cochrane Vascular information specialist, prior to being run [32]. Searches will be performed, in English only, on Medline, Embase, Cochrane (CENTRAL), Web of Science and Google Scholar. Searches for ongoing trials will be repeated via the World Health Organisation International Clinical Trials Registry platform search portal, with supplemental searches as recommended in the Cochrane handbook [31]. All databases will be searched from their inception. No limits will be placed on study language, although a study will be excluded if a translation is not available or feasible to arrange. The reference lists of included studies will be hand searched for further articles. All searches

For peer review only - http://bmjopen.bmj.com/site/about/guidelines.xhtml

will be repeated prior to the end of the meta-analysis to identify any subsequently published literature before the dataset is locked.

Selection of studies

The output of all searches will be imported into Covidence systematic review management software and screened for duplicates [33]. Once duplicates have been removed, two team members will independently screen the list of original papers by title and abstract to identify all RCTs of antithrombotic medication reporting one of the primary outcomes. For identified studies, all additional data sources will be sought utilising focussed searches for further published articles, published letters, trial registry entries, and requests for clinical study reports submitted to medical regulatory authorities. These documents will be bundled and screened in totality against the eligibility criteria independently by two blinded reviewers. The reviewers will attempt to resolve any disagreements once unblinded and a third reviewer will act as a tie breaker where no consensus can be reached.

Data extraction and management

Two team members will extract the data from the full text and supplementary materials of included studies into a pre-piloted data collection form in Covidence developed in collaboration with the statistician. Data on RCT design, participant baseline characteristics, study interventions, methods, all reported study outcomes, results, and the authors' conclusions will be extracted and recorded as detailed in the Cochrane handbook.

Where PAD patients represent a subgroup of the overall study population, data will be collected for both the study population as a whole and the PAD patient subgroup where available. Details of the PAD subgroup including disease status and proportion of non-lower limb atherosclerotic patients will also be recorded. This will permit a sensitivity analysis of the likelihood that using whole-study data rather than subgroup-specific data would introduce bias within the analysis of adverse events (AEs). Where multiple timepoints in follow-up are reported, the longest timepoint shall primarily be used for all outcomes, with subsequent sensitivity analyses exploring the effect of the timepoint of outcome measurement.

Objectives

Primary objectives

1. Define the relative efficacy and hierarchy of efficacy of all antithrombotic medication regimens, previously investigated in RCTs, at reducing the risk of major adverse cardiovascular events (MACE), in patients with PAD.

For peer review only - http://bmjopen.bmj.com/site/about/guidelines.xhtml
For peer review only

2. Define the relative efficacy and hierarchy of efficacy of all antithrombotic medication regimens, previously investigated in RCTs, at reducing the risk of major adverse limb-related events (MALE), in patients with PAD.
3. Define the relative efficacy and hierarchy of efficacy of all antithrombotic medication regimens, previously investigated in RCTs, at reducing the risk of death from any cause, in patients with PAD.
4. Define the relative risk and hierarchy of risk of all antithrombotic medication regimens, previously investigated in RCTs, of serious adverse events (AEs) including fatal bleeding, gastrointestinal bleeding, intracranial bleeding, severe bleeding into any major organ, bleeding requiring blood transfusion and/or return to theatre, and bleeding requiring admission to hospital, in patients with PAD.

Secondary objectives

5. Define the relative risk and hierarchy of risk of all antithrombotic medication regimens, previously investigated in RCTs, of any other recorded adverse drug effects, in patients with PAD.
6. Define the relative compliance to differing antithrombotic medication regimens, previously investigated in RCTs, in patients with PAD.
7. Explore the available data for differential efficacy and/or risks of all antithrombotic medication regimens (objectives 1-4), previously investigated in RCTs, in different subgroups including by gender, age, ethnicity, disease status (asymptomatic/IC/CLTI), conservative versus interventional management, type of interventional management, co-morbidities (other disease states), in patients with PAD.
8. Explore whether any other subgroups of patient or disease characteristics are sufficiently well reported in the included RCTs to establish an analysis and, if so, conduct that subgroup analysis.
9. Establish whether the teams of RCTs that form the judgement forming segments of the networks for the primary objectives are willing to collaborate and have sufficient data to undertake future individual patient network meta-analysis.

Outcomes

Co-primary outcomes:

Co-primary efficacy outcome 1: composite of major adverse cardiovascular events, as defined in available literature, but to include acute coronary syndrome, ischaemic stroke, and cardiovascular death.

For peer review only - http://bmjopen.bmj.com/site/about/guidelines.xhtml

For peer review only

Co-primary efficacy outcome 2: composite of major adverse limb-related events, as defined in available literature, but to include acute limb ischaemia (and embolectomy/thrombectomy/thrombolysis), major amputation (at or above ankle), or need for peripheral revascularisation.

Co-primary efficacy outcome 3: all-cause mortality.

Co-primary safety outcome 4: major bleeding, as defined in available literature, but to include fatal bleeding, symptomatic bleeding in a critical area or organ, such as intracranial, intraspinal, intraocular, retroperitoneal, intra-articular or pericardial, or intramuscular with compartment syndrome, bleeding causing a fall in haemoglobin level of 20 g/L or more, and/or bleeding requiring transfusion of red cells or whole blood.

Secondary outcomes:

Secondary efficacy outcomes will include individual components of MACE and MALE outcomes; cardiovascular death, acute coronary syndrome, ischaemic stroke, major amputation, acute limb ischaemia, thrombectomy/thrombolysis, and need for a subsequent revascularisation procedure.

Secondary safety outcomes will include individual outcomes of fatal bleeding, gastrointestinal bleeding, intracranial bleeding, severe bleeding into any major organ, bleeding requiring blood transfusion and/or return to theatre, and bleeding requiring admission to hospital, venous thromboembolism, rash, discontinuation of assigned therapy for any reason, gastrointestinal symptoms resulting in discontinuation of assigned therapy. Additionally, any further drug-related AEs reported will be included to allow for the identification of unexpected AEs. Adherence to therapy by any quantitative measure will be included, where reported.

Risk of bias assessment

Two study members with domain and meta-analysis expertise will independently assess all included trials for risk of bias using the “ROB 2” tool [34], then areas of uncertainty in these assessments will look to be resolved by reference to collateral information sources as described above (trial registries and regulatory submissions) and contact with the primary investigators if necessary. A risk of bias assessment will be undertaken separately for primary outcome analyses and subgroup analyses.

Measures of treatment effects

For all binary outcomes, the preferred data to collect will be absolute numbers of events and numbers at risk, and risk ratios will be calculated. If the absolute number of events is not available, risk or odds ratios with defined confidence intervals and/or standard error will be the preferred alternative. For continuous outcomes, the mean and standard deviation of the group will be the first choice of measure.

For peer review only - http://bmjopen.bmj.com/site/about/guidelines.xhtml
For peer review only

Unit of analysis issues

Analyses will be conducted at medication regimen level. Dose will not be primarily considered but a secondary sensitivity analysis on the primary outcomes will be undertaken to see if any dose effects are evident. It is not anticipated that any trials will report data in another manner regarding this, however advice will be sought from the statistician should this occur.

Missing data

Only published data will be analysed. Missing data will be considered within the risk of bias assessment. If data is made available through direct correspondence it will be published as an appendix to the review and in the studies data repository.

Transitivity

Recruitment criteria differ between the RCTs and as such the transitivity assumption may be threatened. This is perceived to particularly be the case when considering trials of stable PAD compared with trials of peri-/post-procedural PAD. The validity of the transitivity assumption will be assessed quantitatively by considering the incoherence factor, which involves the comparison of direct and indirect effective estimates for each pair-wise comparison in the network, where they both exist); this will be evaluated using both the local and global strategies detailed in the Cochrane handbook [31].

Assessment of reporting

Aspirin is expected to be the most common comparator within RCTs in this NMA, which will facilitate exploration of publication bias using comparison-adjusted funnel plots [35]. The team has considered selective reporting within trials throughout the study design phase and will qualitatively consider the effect of this during subsequent phases.

Data synthesis

The full analysis plan is available online [36]. Studies will be summarised including the direct comparisons made, population characteristics, and characteristics of those patients with PAD. The medication regimen for both overall and PAD populations will be summarised separately.

Antithrombotic regimens will be grouped into common nodes based on the drugs used in that arm. A network diagram will be constructed for each outcome, where the size of each node is proportional to the number of patients assigned to that intervention, and the thickness of each line is based on the

For peer review only - http://bmjopen.bmj.com/site/about/guidelines.xhtml

inverse of the variance of the direct comparison. Interventions that are absent from a particular network will be highlighted.

Analysis of primary and secondary outcomes will be undertaken on a whole network basis, subject to the checks of assumptions outlined above, wherever networks can be formed based on published data. All NMAs will create pairwise comparisons of medication regimens, and a ranking of all medication regimens will be produced with risk of bias estimates published alongside. Subgroup analyses are discussed further below.

We anticipate a complex NMA based upon prior knowledge. The analysis is planned by the statistician (CH) in R Statistical Software (v4.1) [37], primarily using the *netmeta* package [38]. If the transitivity assumption is violated as the result of differing RCT recruitment criteria, then separate networks will be established to allow meaningful analysis of these data.

Subgroup analysis

The PPI group highlighted the importance of being able to provide individualised recommendations to patients. Therefore, the intent is to extract all published results for subgroups and perform all NMAs that are possible. Anticipated subgroups are PAD disease state (such as IC & CLTI), sex, age, ethnicity, key co-morbidities, and peri-procedural type/status. We also intend to analyse any other subgroups that have sufficient data published but are not anticipated.

Subgroup analyses as described above, will be undertaken wherever sub-networks can be formed based on published data. The inclusion of these sub-analyses will likely be susceptible to non-reporting bias as they are less likely to have formed part of the original RCT per-protocol analysis plan. Candidate interventions that are absent due to non-publication of a particular subgroup analysis will be clearly identified.

Sensitivity analysis

Pre-specified sensitivity analyses, as permitted by available data, will explore consistency in the findings regarding (1) patients with stable versus periprocedural PAD, (2) primary outcomes limited to patients with PAD only (to ascertain whether adverse event rates in trials of mixed atherosclerotic disease e.g., coronary heart disease and stroke, as well as PAD, are applicable to the PAD only population, (3) inclusion of studies at low risk of bias only, (4) restriction of included patients to those with IC and/or CTLI in isolation, (5) primary outcomes by dose of antithrombotic agents (variable dosing strategies, particularly of aspirin, are anticipated to affect adverse event rates), and (6) shorter and longer timepoints (as the optimal antithrombotic regimen may change over time for patients with PAD). Further post-hoc sensitivity analyses may be developed and will be reported transparently as post-hoc in the final report.

For peer review only - http://bmjopen.bmj.com/site/about/guidelines.xhtml
For peer review only

Confidence in cumulative evidence

A summary of findings table will be constructed for the key outcomes, including MACE, MALE, all-cause mortality, major amputation as an individualised outcome, and major bleeding. The underlying quality of evidence for each of these outcomes will be assessed according to the GRADE framework for NMAs, which classifies interventions by both the relative treatment effect size and certainty of evidence [39,40].

Ethics and dissemination

Clinical Impact

The results will be presented to clinical domain specialists at UK national and international vascular and endovascular conferences. Presentations will be made to key stakeholders including NICE, vascular and endovascular societies/charities, and the all-party parliamentary group for vascular and venous disease. The lead applicants' institutions public relations team will maximise the visibility and availability of the results, both to patients and clinicians, through social and traditional media. The results will be disseminated through the UK national societies of vascular specialists and general practitioners to maximise the impact on clinical practice. The authors anticipate that the results of the research would be sufficient to trigger a review of the current NICE guidelines.

Patient Impact

The patient accessible report co developed with the PPI group will be submitted to UK vascular disease charities, such as the Circulation Foundation and the British Heart Foundation, for publication on their websites.

Data storage

The data generated from this research will be an essential output. The data collected from screening, risk assessments, study results, and statistical coding will be made available on the lead applicants institution research data repository to ensure ongoing future access. This will facilitate research efficiency if/when future trials become available, and the analysis requires updating. We will seek to establish a resource that can be added-to, re-evaluated, or re-analysed with future trail data. A link to the data repository will be provided in the PROSPERO record for the systematic review.

Publications

The authors anticipate that the primary paper will be published, open access, in a high impact cardiovascular journal. Any further publications will also be submitted, open access.

For peer review only - http://bmjopen.bmj.com/site/about/guidelines.xhtml

Acknowledgements

The authors thank Candida Fenton from the Cochrane Vascular Group, University of Edinburgh, Edinburgh, United Kingdom, for peer review of the search strategy.

Funding:

This project is funded by the National Institute for Health and Care Research (NIHR) under its Research for Patient Benefit (RfPB) Programme (Grant Reference Number NIHR204123). The views expressed are those of the author(s) and not necessarily those of the NIHR or the Department of Health and Social Care.

Competing interest statement: We have read and understood the BMJ Group policy on declaration of interests and declare the following interests: Nil - Except above funding.

Contributor statement: All authors drafted and revised the manuscript. Mr Iain N Roy is the guarantor for this work. The corresponding author attests that all listed authors meet authorship criteria and that no others meeting the criteria have been omitted.

For peer review only

For peer review only - http://bmjopen.bmj.com/site/about/guidelines.xhtml

References

1 Morley RL, Sharma A, Horsch AD, *et al.* Peripheral artery disease. *BMJ* 2018;**360**:j5842. doi:10.1136/bmj.j5842

2 Conte MS, Pomposelli FB, Clair DG, *et al.* Society for Vascular Surgery practice guidelines for atherosclerotic occlusive disease of the lower extremities: Management of asymptomatic disease and claudication. *J Vasc Surg* 2015;**61**:2S-41S.e1. doi:10.1016/j.jvs.2014.12.009

3 Aboyans V, Ricco JB, Bartelink MLEL, *et al.* 2017 ESC Guidelines on the Diagnosis and Treatment of Peripheral Arterial Diseases, in collaboration with the European Society for Vascular Surgery (ESVS). *Eur Heart J* 2018;**39**:763–816. doi:10.1093/eurheartj/ehx095

4 Farber A. Chronic Limb-Threatening Ischemia. *New England Journal of Medicine* 2018;**379**:171–80. doi:10.1056/nejmcp1709326

5 Song P, Rudan D, Zhu Y, *et al.* Global, regional, and national prevalence and risk factors for peripheral artery disease in 2015: an updated systematic review and analysis. *Lancet Glob Health* 2019;**7**:e1020–30. doi:10.1016/S2214-109X(19)30255-4

6 Criqui MH, Aboyans V. Epidemiology of Peripheral Artery Disease. *Circ Res* 2015;**116**:1509–26. doi:10.1161/CIRCRESAHA.116.303849

7 Institute for Health Metrics and Evaluation (IHME). Global Burden of Disease. 2019.https://ghdx.healthdata.org (accessed 8 Apr 2022).

8 National Clinical Guideline Centre (UK). *Lower Limb Peripheral Arterial Disease: Diagnosis and Management [Internet]*. London: Royal College of Physicians (UK): : National Institute for Health and Clinical Excellence: Guidance 2012. https://www.ncbi.nlm.nih.gov/books/NBK299071/

9 Shishehbor MH, White CJ, Gray BH, *et al.* Critical Limb Ischemia. *J Am Coll Cardiol* 2016;**68**:2002–15. doi:10.1016/j.jacc.2016.04.071

10 Gerhard-Herman MD, Gornik HL, Barrett C, *et al.* 2016 AHA/ACC Guideline on the Management of Patients With Lower Extremity Peripheral Artery Disease: Executive Summary: A Report of the American College of Cardiology/American Heart Association Task Force on Clinical Practice Guidelines. *J Am Coll Cardiol* 2017;**69**:1465–508. doi:10.1016/j.jacc.2016.11.008

11 Cea-Soriano L, Fowkes FGR, Johansson S, *et al.* Time trends in peripheral artery disease incidence, prevalence and secondary preventive therapy: A cohort study in the Health Improvement Network in the UK. *BMJ Open* 2018;**8**:1–7. doi:10.1136/bmjopen-2017-018184

12 Foley TR, Armstrong EJ, Waldo SW. Contemporary evaluation and management of lower extremity peripheral artery disease. *Heart* 2016;**102**:1436–41. doi:10.1136/HEARTJNL-2015-309076

13 National Institute for Health and Care Excellence. Peripheral arterial disease: diagnosis and management. Clinical guideline [CG147]. 2012.https://www.nice.org.uk/guidance/cg147 (accessed 20 Apr 2022).

For peer review only - http://bmjopen.bmj.com/site/about/guidelines.xhtml

14 Anand SS, Widimsky P, Alings M, *et al.* Rivaroxaban with or without aspirin in patients with stable peripheral or carotid artery disease: an international, randomised, double-blind, placebo-controlled trial. *The Lancet (British edition)* 2018;**391**:219–29. doi:10.1016/S0140-6736(17)32409-1

15 Eikelboom JW, Connolly SJ, Bosch J, *et al.* Rivaroxaban with or without aspirin in stable cardiovascular disease. *New England Journal of Medicine* 2017;**377**:1319–30. doi:10.1056/NEJMoa1709118

16 CAPRIE Steering Committee. A randomised, blinded, trial of clopidogrel versus aspirin in patients at risk of ischaemic events (CAPRIE). *Lancet* 1996;**348**:1329–39. doi:10.1016/s0140-6736(96)09457-3

17 Becquemin J-P. Effect of Ticlopidine on the Long-Term Patency of Saphenous-Vein Bypass Grafts in the Legs. *N Engl J Med* 1997;**337**:1726–31. doi:10.1056/NEJM199712113372404

18 Hiatt WR, Fowkes FGR, Heizer G, *et al.* Ticagrelor versus Clopidogrel in Symptomatic Peripheral Artery Disease. *N Engl J Med* 2017;**376**:32–40. doi:10.1056/NEJMoa1611688

19 Bonaca MP, Bauersachs RM, Anand SS, *et al.* Rivaroxaban in Peripheral Artery Disease after Revascularization. *N Engl J Med* 2020;**382**:1994–2004. doi:10.1056/NEJMoa2000052

20 Strobl FF, Brechtel K, Schmehl J, *et al.* Twelve-month results of a randomized trial comparing mono with dual antiplatelet therapy in endovascularly treated patients with peripheral artery disease. *J Endovasc Ther* 2013;**20**:699–706. doi:10.1583/13-4275MR.1

21 Burdess A, Nimmo AF, Garden OJ, *et al.* Randomized controlled trial of dual antiplatelet therapy in patients undergoing surgery for critical limb ischemia. *Ann Surg* 2010;**252**:37–42. doi:10.1097/SLA.0b013e3181e40dde

22 Warfarin Antiplatelet Vascular Evaluation Trial Investigators, Anand S, Yusuf S, *et al.* Oral anticoagulant and antiplatelet therapy and peripheral arterial disease. *N Engl J Med* 2007;**357**:217–27. doi:10.1056/NEJMoa065959

23 National Institute for Health and Care Excellence. Clopidogrel and modified-release dipyridamole for the prevention of occlusive vascular events. Technology appraisal guidance [TA210]. 2010.https://www.nice.org.uk/guidance/ta210 (accessed 20 Apr 2022).

24 National Institute for Health and Care Excellence. Rivaroxaban for preventing atherothrombotic events in people with coronary or peripheral artery disease. Technology appraisal guidance [TA607]. 2019.https://www.nice.org.uk/guidance/ta607 (accessed 20 Apr 2022).

[dataset] 25 Roy I, Sidebottom D. Protocol - Systematic review & network meta-analysis of randomised controlled trials comparing antithrombotic drugs for cardiovascular risk reduction in patients with lower limb peripheral arterial disease. 2022. doi:10.24376/rd.sgul.21591216.v1

26 Roy I, Sidebottom DB, Holt P, *et al.* Antithrombotic drugs for cardiovascular risk reduction in patients with lower limb peripheral arterial disease: protocol for a systematic review and network meta-analysis of randomised controlled trials. PROSPERO 2023. https://www.crd.york.ac.uk/prospero/display_record.php?ID=CRD42023389262 (accessed 5 Jan 2023).

For peer review only - http://bmjopen.bmj.com/site/about/guidelines.xhtml

27 Page MJ, McKenzie JE, Bossuyt PM, *et al.* The PRISMA 2020 statement: an updated guideline for reporting systematic reviews. *BMJ* 2021;**372**:n71. doi:10.1136/bmj.n71

28 Rethlefsen ML, Kirtley S, Waffenschmidt S, *et al.* PRISMA-S: an extension to the PRISMA Statement for Reporting Literature Searches in Systematic Reviews. *Syst Rev* 2021;**10**:39. doi:10.1186/s13643-020-01542-z

29 Hutton B, Salanti G, Caldwell DM, *et al.* The PRISMA extension statement for reporting of systematic reviews incorporating network meta-analyses of health care interventions: checklist and explanations. *Ann Intern Med* 2015;**162**:777–84. doi:10.7326/M14-2385

30 Joint Formulary Committee. British National Formulary (online). 2022.http://www.medicinescomplete.com (accessed 20 Apr 2022).

31 Higgins J, Thomas J, Chandler J, *et al.* Cochrane Handbook for Systematic Reviews of Interventions. Cochrane Handbook for Systematic Reviews of Interventions version 6.3 (updated Feburary 2022). 2022.https://training.cochrane.org/handbook (accessed 20 Apr 2022).

[dataset] 32 Roy I, Sidebottom DB, John-Pierre K. Search strategy - Systematic review & network meta-analysis of randomised controlled trials comparing antithrombotic drugs for cardiovascular risk reduction in patients with lower limb peripheral arterial disease. 2023. doi:10.24376/rd.sgul.21687866

33 Veritas Health Innovation. Covidence systematic review software. 2022.www.covidence.org (accessed 20 Apr 2022).

34 Sterne JAC, Savović J, Page MJ, *et al.* RoB 2: a revised tool for assessing risk of bias in randomised trials. *BMJ* 2019;**366**:l4898. doi:10.1136/bmj.l4898

35 Chaimani A, Salanti G. Using network meta-analysis to evaluate the existence of small-study effects in a network of interventions. *Res Synth Methods* 2012;**3**:161–76. doi:10.1002/jrsm.57

[dataset] 36 Roy I, Huang C. Analysis Plan - Systematic review & network meta-analysis of randomised controlled trials comparing antithrombotic drugs for cardiovascular risk reduction in patients with lower limb peripheral arterial disease. 2023. doi:10.24376/rd.sgul.21688040

37 R Core Team. R: A language and environment for statistical computing. 2022.

38 Rücker G, Krahn U, König J, *et al.* netmeta: Network Meta-Analysis using Frequentist Methods. 2022.https://cran.r-project.org/package=netmeta

39 Brignardello-Petersen R, Izcovich A, Rochwerg B, *et al.* GRADE approach to drawing conclusions from a network meta-analysis using a partially contextualised framework. *The BMJ* 2020;**371**. doi:10.1136/bmj.m3907

40 Brignardello-Petersen R, Florez ID, Izcovich A, *et al.* GRADE approach to drawing conclusions from a network meta-analysis using a minimally contextualised framework. *The BMJ* 2020;**371**:1–8. doi:10.1136/bmj.m3900

For peer review only

For peer review only - http://bmjopen.bmj.com/site/about/guidelines.xhtml

Supplemental Material

Antithrombotic medications currently named in the British National Formulary are aspirin, clopidogrel, dipyridamole, prasugrel, ticagrelor, cangrelor, warfarin sodium, acenocoumarol, phenindione, apixaban, edoxaban, rivaroxaban, dabigatran etexilate, heparin (unfractionated), dalteparin sodium, enoxaparin sodium, tinzaparin sodium, danaparoid sodium, argatroban monohydrate, bivalirudin, epoprostenol, and fondaparinux.ubmission

For peer review only

PRISMA-P 2015 Checklist

This checklist has been adapted for use with protocol submissions to *Systematic Reviews* from Table 3 in Moher D et al: Preferred reporting items for systematic review and meta-analysis protocols (PRISMA-P) 2015 statement. *Systematic Reviews* 2015 **4**:1

Section/topic	#	Checklist item	Information reported Yes	Information reported No	Line number(s)
**ADMINISTRATIVE INFORMATION**					
**Title**					
Identification	1a	Identify the report as a protocol of a systematic review	☒	☐	1:1-4
Update	1b	If the protocol is for an update of a previous systematic review, identify as such	☐	☒	N/A
**Registration**	2	If registered, provide the name of the registry (e.g., PROSPERO) and registration number in the Abstract	☒	☐	2:37
**Authors**					
Contact	3a	Provide name, institutional affiliation, and e-mail address of all protocol authors; provide physical mailing address of corresponding author	☒	☐	1:10-25
Contributions	3b	Describe contributions of protocol authors and identify the guarantor of the review	☒	☐	1:44-46
**Amendments**	4	If the protocol represents an amendment of a previously completed or published protocol, identify as such and list changes; otherwise, state plan for documenting important protocol amendments	☐	☒	N/A
**Support**					
Sources	5a	Indicate sources of financial or other support for the review	☒	☐	1:30-33
Sponsor	5b	Provide name for the review funder and/or sponsor	☒	☐	1:30-35
Role of sponsor/funder	5c	Describe roles of funder(s), sponsor(s), and/or institution(s), if any, in developing the protocol	☒	☐	1:37-39
**INTRODUCTION**					
**Rationale**	6	Describe the rationale for the review in the context of what is already known	☒	☐	4:1-47
**Objectives**	7	Provide an explicit statement of the question(s) the review will address with reference to participants, interventions, comparators, and outcomes (PICO)	☒	☐	4:51-54
**METHODS**					

For peer review only - http://bmjopen.bmj.com/site/about/guidelines.xhtml

Section/topic	#	Checklist item	Information reported Yes	No	Line number(s)
**Eligibility criteria**	8	Specify the study characteristics (e.g., PICO, study design, setting, time frame) and report characteristics (e.g., years considered, language, publication status) to be used as criteria for eligibility for the review	☒	☐	5:1-41
**Information sources**	9	Describe all intended information sources (e.g., electronic databases, contact with study authors, trial registers, or other grey literature sources) with planned dates of coverage	☒	☐	6:15-23
**Search strategy**	10	Present draft of search strategy to be used for at least one electronic database, including planned limits, such that it could be repeated	☒	☐	6:13 (cited)
STUDY RECORDS					
Data management	11a	Describe the mechanism(s) that will be used to manage records and data throughout the review	☒	☐	6:25-35
Selection process	11b	State the process that will be used for selecting studies (e.g., two independent reviewers) through each phase of the review (i.e., screening, eligibility, and inclusion in meta-analysis)	☒	☐	6:28-43
Data collection process	11c	Describe planned method of extracting data from reports (e.g., piloting forms, done independently, in duplicate), any processes for obtaining and confirming data from investigators	☒	☐	6:39-52
**Data items**	12	List and define all variables for which data will be sought (e.g., PICO items, funding sources), any pre-planned data assumptions and simplifications	☒	☐	6:41-52
**Outcomes and prioritization**	13	List and define all outcomes for which data will be sought, including prioritization of main and additional outcomes, with rationale	☒	☐	7:33-8:11
**Risk of bias in individual studies**	14	Describe anticipated methods for assessing risk of bias of individual studies, including whether this will be done at the outcome or study level, or both; state how this information will be used in data synthesis	☒	☐	8:13-19
DATA					
**Synthesis**	15a	Describe criteria under which study data will be quantitatively synthesized	☒	☐	9:1 (cited), 11-20
	15b	If data are appropriate for quantitative synthesis, describe planned summary measures, methods of handling data, and methods of combining data from studies, including any planned exploration of consistency (e.g., I^2, Kendall's tau)	☒	☐	8:41-48, 9:1 (cited) - 20
	15c	Describe any proposed additional analyses (e.g., sensitivity or subgroup analyses, meta-regression)	☒	☐	9:22-34
	15d	If quantitative synthesis is not appropriate, describe the type of summary planned	☒	☐	9:18-20
**Meta-bias(es)**	16	Specify any planned assessment of meta-bias(es) (e.g., publication bias across studies, selective reporting within studies)	☒	☐	8:50-55
**Confidence in cumulative evidence**	17	Describe how the strength of the body of evidence will be assessed (e.g., GRADE)	☒	☐	9:49-54

For peer review only

For peer review only - http://bmjopen.bmj.com/site/about/guidelines.xhtml
